# High levels of FLT3-ligand in bone marrow and peripheral blood of patients with advanced multiple myeloma

**DOI:** 10.1371/journal.pone.0181487

**Published:** 2017-07-20

**Authors:** Normann Steiner, Roman Hajek, Sabina Sevcikova, Bojana Borjan, Karin Jöhrer, Georg Göbel, Gerold Untergasser, Eberhard Gunsilius

**Affiliations:** 1 Department of Internal Medicine V (Hematology and Medical Oncology), Innsbruck Medical University, Anichstr. 35, Innsbruck, Austria; 2 Laboratory for Tumor Biology & Angiogenesis, Innsbruck Medical University, Innrain 66, Innsbruck, Austria; 3 Faculty of Medicine, University of Ostrava, Ostrava, Czech Republic; 4 Department of Hematology-Oncology, University Hospital Ostrava, Ostrava, Czech Republic; 5 Babak Myeloma Group, Department of Pathological Physiology, Faculty of Medicine, Masaryk University, Brno, Czech Republic; 6 Department of Clinical Hematology, University Hospital Brno, Czech Republic; 7 Tyrolean Cancer Research Institute, Innrain 66, Innsbruck, Austria; 8 Department of Medical Statistics, Informatics and Health Economics, Innsbruck Medical University, Schöpfstr. 41, Innsbruck, Austria; Institut national de la recherche scientifique, CANADA

## Abstract

**Introduction:**

Multiple myeloma (MM) is still incurable due to resistance against various therapies. Thus, the identification of biomarkers predicting progression is urgently needed. Here, we evaluated four biomarkers in bone marrow and peripheral blood of MM patients for their prognostic significance.

**Materials & methods:**

Bone marrow- and peripheral blood plasma levels of FLT3-L, soluble TIE2, endostatin, and osteoactivin were determined in patients with monoclonal gammopathy of undetermined significance (MGUS, n = 14/n = 4), patients with newly diagnosed MM (NDMM, n = 42/n = 31) and patients with relapsed/refractory MM (RRMM, n = 27/n = 16) by sandwich ELISA.

**Results:**

Median FLT3-L expression increased from MGUS (58.77 pg/ml in bone marrow; 80.40 pg/ml in peripheral blood) to NDMM (63.15 pg/ml in bone marrow; 85.05 pg/ml in peripheral blood) and was maximal in RRMM (122 pg/ml in bone marrow; 160.47 pg/ml in peripheral blood; NDMM vs. RRMM p<0.001). A cut-off value of FLT3-L >92 pg/ml in bone marrow and >121 pg/ml in peripheral blood was associated with relapse or refractoriness in MM patients. FLT3-L was found to be a high predictive marker for discrimination between NDMM and RRMM as well in bone marrow as in peripheral blood (AUC 0.75 in bone marrow; vs 0.84 in peripheral blood).

**Conclusion:**

High levels of FLT3-L in bone marrow and peripheral blood of MM patients identify patients with progressive disease and are associated with relapse or refractoriness in MM patients. FLT3-L could be useful as a marker to identify RRMM patients and should be evaluated as target for future therapies.

## Introduction

Multiple myeloma (MM) remains an incurable disease despite the implementation of new substances such as lenalidomide, pomalidomide, carfilzomib or new antibodies into the treatment. Although the overall and progression free survival has improved considerably due to modern therapies [1–3], genetic alterations in MM cells and the bone marrow microenvironment are responsible for resistance to treatments [4]. Among the therapeutic armentarium against MM there are different antiangiogenic agents, e.g. thalidomide, lenalidomide, and pomalidomide. The interaction between bone marrow microenvironment and MM cells plays a central role for MM progression. Formation of new blood vessels is regulated by pro- and anti-angiogenic factors. Vacca and co-workers described an “angiogenic switch” from MGUS to active MM due to an overproduction of angiogenic cyto- and chemokines by MM cells [5]. Several cells (stromal cells, osteoblasts, osteoclasts, endothelial cells, adipocytes, T-cells, natural killer cells) and mediators (adhesion molecules, cytokines, growth factors) participate in MM angiogenic processes and its progression [6, 7]. Hitherto, no reliable biomarkers are available for predicting therapy response and disease progression.

Angiopoietins (Ang-1 and Ang-2) and their receptor TIE2 are involved in process of angiogenesis. Increased levels of Ang-2 are known to correlate with progression in MM [8–10]. The tyrosine kinase receptor TIE2 is located on vascular endothelial cells and activated TIE2 is able to support their survival and adhesion, resulting in the stabilization of blood vessels [11]. TIE2 also appears to regulate hematopoietic stem cell quiescence in the bone marrow stem cell niche [12]. Moreover, TIE2 is expressed on leukemia cells and several solid tumors, hence, the receptor may be an attractive target for cancer therapy [13]. Soluble TIE2 has been shown to inhibit angiopoietin-mediated TIE2 activation and downstream signaling by binding Ang-1 and Ang-2. Therefore, soluble TIE2 promotes angiogenesis and vascular remodeling by inhibiting vascular stabilization [14].

Fms–like tyrosine kinase 3 (FLT3), a receptor-type tyrosin-protein kinase class III, is located on the surface of hematopoietic progenitor cells. The cytokine FLT3-ligand (FLT3-L) can be produced by endothelial cells as a membrane-bound and as soluble form [15] promoting progenitor cell growth and differentiation [16, 17]. Soluble FLT3-L seems to have a positive correlation with the angiogenic process of multiple myeloma [18].

Endostatin, a terminal fragment of type XVIII collagen, is an endogenous inhibitor of angiogenesis [19–21]. It interacts with endothelial cell molecules, leads to stabilization of blood vessels and, consequently, to inhibition of tumor growth [22].

Osteoactivin, a regulator of osteoblast differentiation, is responsible for bone formation in physiological and pathological conditions [23, 24] and inhibits osteoclastogenesis mediated through CD44-ERK signaling [25]. A previous study associated osteoactivin with promotion of angiogenesis after shedding its ectodomain of the surface of breast cancer cells [26]. Moreover, it has been shown that osteoactivin acts as communicating molecule promoting osteogenesis and angiogenesis [27]. The role of osteoactivin as a potential angiogenic factor in multiple myeloma is still not defined.

Hitherto, no reports about soluble TIE2-, FLT3-L-, endostatin- and osteoactivin- levels in different stages of MM disease are available. The aim of our study was to evaluate these factors in bone marrow aspirates and peripheral blood plasma of patients with monoclonal gammopathy of undetermined significance (MGUS), patients with newly diagnosed MM (NDMM) and patients with relapsed/refractory MM (RRMM) for their prognostic significance.

## Materials and methods

### Ethics statement

Investigations have been conducted in accordance with the Declaration of Helsinki and the national and international guidelines and have been approved by the authors’ institutional review board (number: AN2015-0034 346/4.13; AN5064 Innsbruck Innsbruck and 20/1/2011; Brno). Patients who provided written informed consent were enrolled.

### Patients and sample collection

Patients with monoclonal gammopathy of undetermined significance (MGUS, n = 14), newly diagnosed MM (NDMM, n = 42) and relapsed/refractory MM (RRMM, n = 27) according to the International Myeloma Working Group (IMWG) criteria [28], were included in the study population. All relevant clinical data and disease characteristics are shown in (Table 1). Bone marrow aspirates from MGUS- (n = 14), NDMM- (n = 42), RRMM patients (n = 27), the related peripheral blood from MGUS- (n = 4), NDMM- (n = 31), and RRMM patients (n = 16) and peripheral blood from control persons (n = 16) underwent centrifugation for 10 min at 1000 x *g* and obtained bone marrow and peripheral blood plasma was collected and stored at -80°C.

**Table 1:** Patient demographics and characteristics (n = 73).

Parameter	MGUS		NDMM		RRMM	
	n = 14	%	n = 42	%	n = 26	%
Median age (range), years	64 (34–81)		71 (45–89)		62 (36–78)	
Sex f/m						
F	4	29	20	48	13	50
M	10	71	22	52	13	50
ISS						
I			11	26	8	30
II			8	19	9	35
III			23	55	9	35
Type of Ig heavy chain (serum)						
IgG	12	86	23	55	18	69
IgM	0	0	0	0	2	8
IgA	2	14	8	19	2	8
IgD	0	0	1	2	0	0
Light chain only	0	0	10	24	4	15
Type of Ig light chain (serum)						
Kappa	7	50	26	62	15	58
Lambda	7	50	16	38	11	42
β-2 microglobulin >UNV	8	62	32	82	19	73
LDH >UNV	4	29	10	24	7	27
Creatinine ≥1.3 mg/dl	10	71	26	62	12	44
Serum calcium >UNV	0	0	8	19	2	8
Hemoglobin ≤12 g/dl	7	50	34	81	18	69
Platelets <100,000/mm^3^	2	14	8	20	12	46
Osteolytic bone lesions	1	7	33	79	26	100
Cytogenetic standard risk	4	29	22	52	5	19
Cytogenetic high risk	1	7	15	36	14	54
Cytogenetic not available	9	64	5	12	7	27
Therapy lines at samples collection						
1^st^ line					0	0
2^nd^ line					5	19
3^rd^ line					10	39
4^th^ line					4	15
5^th^ line					2	8
6^th^ line					4	15
7^th^ line					1	4
BTZ based therapy					20	77
IMiD based therapy					6	23

N, number of patients; ISS, International staging system; Ig, Immunoglobulin; UNV, upper normal value; LDH, lactatedehydrogenasis; IMiD, Immunomodulatory drugs; BTZ, Bortezomib

### Sandwich ELISA for soluble TIE2, FLT3-L, endostatin, and osteoactivin

To quantify levels of soluble TIE2, FLT3-L, endostatin, and osteoactivin in bone marrow- and peripheral blood plasma samples from the subjects in the 3 cohorts and the control persons, commercially available solid-phase human TIE2, FLT3-L, endostatin and osteoactivin ELISA kits, all purchased from ThermoFisher Scientific, were used according to the manufacturer’s instructions. Briefly, plasma samples, diluted 3-fold with the assay buffer, were incubated in duplicates in the ELISA plate pre-coated with specific capture antibody for 2.5 hours at room temperature. Four washes were performed to remove unbound proteins and each well was incubated with biotin labeled specific detection antibody at room temperature for 1 hour. After repeated washing steps and removal of unbound antibody, the plate was treated with a streptavidin-horseradish peroxidase conjugate for 45 min. Following an enzymatic reaction with the substrate for peroxidase (room temperature, 30 min) and subsequent termination of the color development, the absorbance at 450 nm and 550 nm was measured using an automated microplate reader.

### Statistical analysis

Descriptive data are shown using median and interquartile range. We used mainly non-parametric tests (Wilcoxon test, Kruskal-Wallis test) to identify differences between groups. Pearson correlation coefficient was used for associations between quantitative variables. Sensitivity, specifity, predictive values and thresholds were calculated by ROC analysis and Area under the Curves (AUCs) using Youden's approach. All tests for statistical significance were two-sided. A p-value less than 0.05 was considered as statistically significant. Statistical evaluation was performed using SPSS statistical software (version 21.0; SPSS Inc., Chicago, IL, USA).

## Results

### Soluble TIE2 levels in bone marrow differ significantly in patients with MGUS, NDMM and RRMM

**Fig 1:**
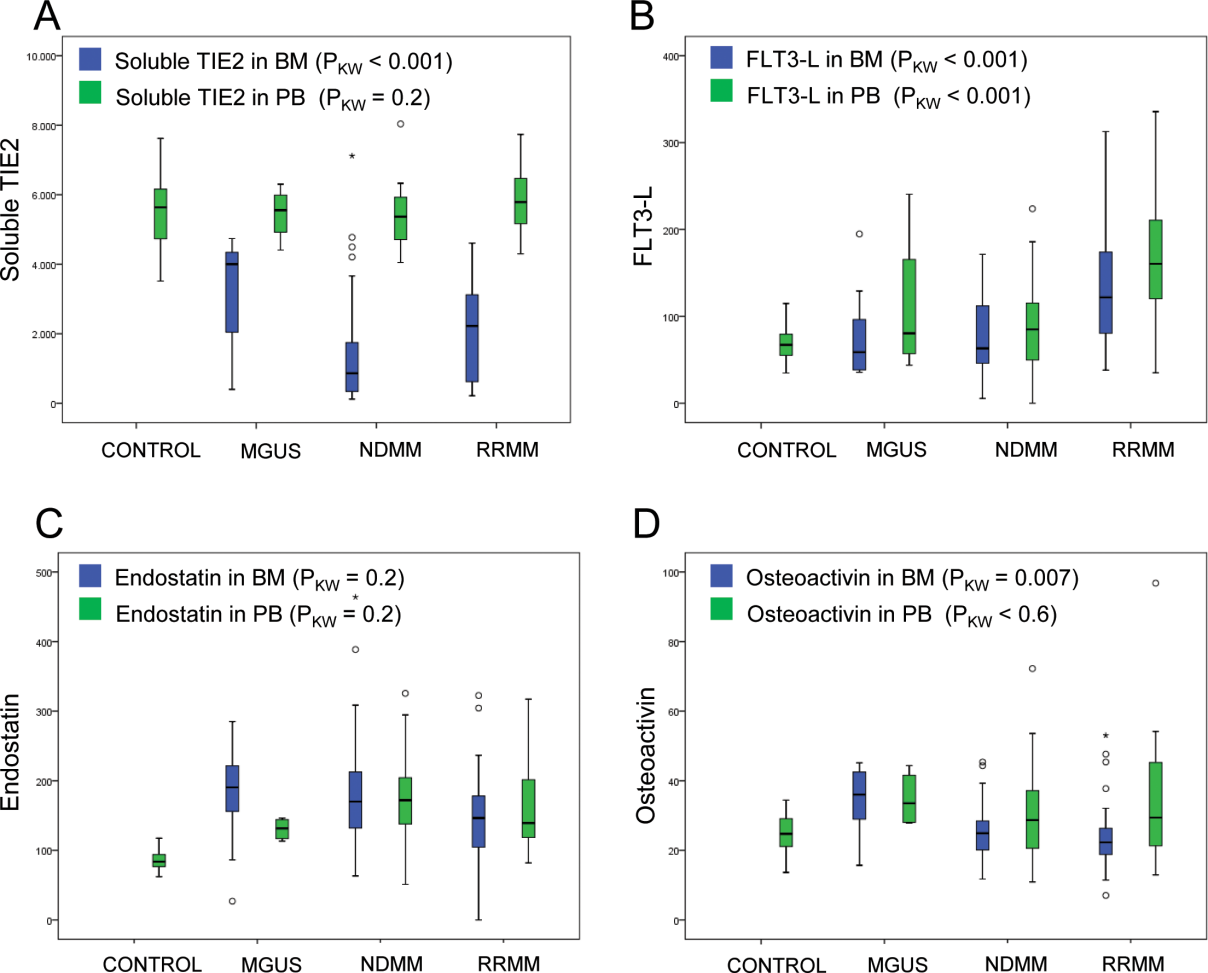
Expression of soluble TIE2, FLT3-L, endostatin, and osteoactivin in bone marrow and peripheral blood of MGUS, NDMM and RRMM patients. (A) Soluble TIE2. (B) FLT3-L. (C) Endostatin. (D) Osteoactivin.

In the three cohorts, expression of soluble TIE2 in bone marrow differs significantly between MGUS-, NDMM- and RRMM patients (p<0.001; Fig 1A). Highest soluble TIE2 expression in bone marrow was seen in MGUS patients (median 4003.97 pg/ml) in comparison to RRMM- (median 2223.26 pg/m; p = 0.03) and to NDMM patients (median 861.98 pg/ml; p<0.001). Statistical significant difference between bone marrow levels of soluble TIE2 was observed for RRMM- and NDMM patients (p = 0.03; Table 2A). Expression of soluble TIE2 in peripheral blood plasma did not differ significantly in the three cohorts (p = 0.2; Fig 1A). Measurements of soluble TIE2 in bone marrow and peripheral blood correlated significantly (ρ 0.53; p<0.001). We did not found significant differences of soluble TIE2 levels between patients and control person in peripheral blood (p>0.9).

**Table 2:** Soluble TIE2 and FLT3-L expression levels from bone marrow (BM) and peripheral blood (PB) correlated with MGUS, NDMM and RRMM patients.

**A** Patients	**Soluble TIE2**	**Soluble TIE2**	**Soluble TIE2**	
	**Median (pg/ml)**	**[95% CI]**	**IQR**	**p-value**
Control P (n = 16) MGUS BM (n = 14) MGUS PB (n = 4)	5638.37 4003.97 5556.47	4799.26–6095.14 2045.33–4345.15 4408.60–6302.80	4706.64–6245.47 1674.58–4397.41 4666.05–6145.73	
NDMM BM (n = 42)	861.98	393.22–1533.42	341.46–1829.64	
NDMM PB (n = 31)	5368.25	4938.70–5669.45	4644.90–5962.00	
RRMM BM (n = 27)	2223.26	740.09–2919.40	562.43–3148.80	
RRMM PB (n = 16)	5787.12	5166.65–6470.97	5120.22–6496.36	
All patients BM (83) All patients PB (51)	1353.05 5599.50	739.59–2223.26 5163.23–5744.85	430.27–3386.15 4867.20–5998.45	P<0.001 P = 0.2
**B** Patients	**FLT3-L**	**FLT3-L**	**FLT3-L**	
	**median (pg/ml)**	**[95% CI]**	**IQR**	**p-value**
Control P (n = 16) MGUS BM (n = 14) MGUS PB (n = 4)	67.17 58.77 80.04	55.52–76.40 38.35–96.35 43.80–240.40	55.03–80.98 38.23–101.62 50.38–202.96	
NDMM BM (n = 42)	63.15	52.48–82.74	45.11–114.08	
NDMM PB (n = 31)	85.05	61.92–103.90	45.25–116.70	
RRMM BM (n = 27)	122.00	93.80–140.77	80.25–174.90	
RRMM PB (n = 16)	160.47	124–199.05	118.37–216.41	
All patients BM (83) All patients BP (51)	77.25 96.40	63.0–100.45 85.05–119.30	48.75–128.19 62.15–158.85	P<0.001 P<0.001

MGUS, monoclonal gammopathy undetermined significance; MM, multiple

Myeloma; NDMM, newly diagnosed multiple myeloma; RRMM, relapsed refractory multiple myeloma; [95% CI], Confidence interval; IQR, Interquartile range; Control P, control person

### FLT3-L levels are significantly elevated in bone marrow- and peripheral blood plasma of patients with RRMM

The cytokine FLT3-ligand (FLT3-L) showed significant differences of expression in bone marrow and peripheral blood of MGUS-, NDMM- and RRMM patients (in bone marrow p<0.001; in peripheral blood p<0.001, Fig 1B). RRMM patients had significantly higher FLT3-L levels (median 122 pg/ml in bone marrow; median 160.47 pg/ml in peripheral blood) in comparison to MGUS- (median 58.77 in bone marrow; median 80.04 pg/ml in peripheral blood; p<0,001) and NDMM patients (median 63.15 pg/ml in bone marrow; median 85.05 pg/ml in peripheral blood; p<0,001; Table 2B). Measurements of FLT3-L in bone marrow and peripheral blood correlated significantly (ρ 0.68; p<0.001). Additionally, we found significant differences of FLT3-L levels between patients and control person in peripheral blood (p = 0.02).

### Endostatin levels are reduced in bone marrow plasma of patients with RRMM

Endostatin in plasma of bone marrow showed, in contrast to TIE2 and FLT3-L, the lowest expression in RRMM patients (median 146.50 ng/ml). Higher levels of endostatin were found in MGUS patients (median 190.37 mg/dl) compared to NDMM- (median 170.15 mg/ml, p = 0.5) and RRMM patients (median 146.50 ng/ml; p = 0.08; Fig 1C, Table 3A). Plasma levels of endostatin in peripheral blood did not differ significantly in the three cohorts (p = 0.2). Measurements of endostatin in bone marrow and peripheral blood correlated significantly (ρ 0.71; p<0.001). We found significant differences of endostatin levels between patients and control person in peripheral blood (p<0.001).

**Table 3:** Endostatin and osteoactivin expression levels from bone marrow (BM) and peripheral blood (PB) correlated with MGUS, NDMM and RRMM patients.

**A** Patients	**Endostatin**	**Endostatin**	**Endostatin**	
	**median (ng/ml)**	**[95% CI]**	**IQR**	**p-value**
Control P (n = 16) MGUS BM (n = 14) MGUS PB (n = 4)	83.70 190.37 131.55	77.35–92.60 156.00–221.55 113.05–146.45	76.37–94.70 147.52–222.77 115.01–145.38	
NDMM BM (n = 42)	170.15	144.45–198.05	130.07–213.10	
NDMM PB (n = 31)	172.05	148.85–191.18	132.45–205.95	
RRMM BM (n = 27)	146.50	121.85–169.55	100.14–181.63	
RRMM PB (n = 16)	139.17	119.55–190.90	118.05–206.95	
All patients BM (83) All patients PB (51)	165.40 159.05	146.50–185.25 142.20–175.45	124.25–209.30 119.55–202.90	P = 0.2 P = 0.2
**B** Patients	**Osteoactivin**	**Osteoactivin**	**Osteoactivin**	
	**median (ng/ml)**	**[95% CI]**	**IQR**	**p-value**
Control P (n = 16) MGUS BM (n = 14) MGUS PB (n = 4)	24.74 36.02 33.55	21.82–29.12 28.95–42.52 27.85–44.35	20.71–29.12 27.25–42.67 27.95–42.97	
NDMM BM (n = 42)	24.92	21.55–27.04	19.83–28.62	
NDMM PB (n = 31)	28.70	25.01–34.70	19.35–37.80	
RRMM BM (n = 27)	22.30	20.05–25.75	18.30–26.50	
RRMM PB (n = 16)	29.45	23.47–44.21	20.23–47.40	
All patients BM (83) All patients PB (51)	24.80 28.80	22.15–26.85 26.60–35.95	20.10–33.30 21.80–40.20	P = 0.007 P<0.6

MGUS, monoclonal gammopathy undetermined significance; MM, multiple

Myeloma; NDMM, newly diagnosed multiple myeloma; RRMM, relapsed refractory

multiple myeloma; [95% CI], Confidence interval; IQR, Interquartile range; Control P, control person

### Osteoactivin levels differ between bone marrow plasma of MGUS, NDMM and RRMM patients

In bone marrow plasma of MGUS patients a significantly higher osteoactivin expression (median 36 ng/ml) in bone marrow in comparison to NDMM- (median 24.92 ng/ml; p = 0.004) and RRMM patients (median 22.30 ng/ml; p = 0.006; Fig 1D) was found. Low expression of osteoactivin was observed in bone marrow plasma of NDMM- (median 24.92 ng/ml) and RRMM patients (median 22.30 ng/ml; Table 3B). Osteoactivin levels in peripheral blood did not differ significantly in the three cohorts (p<0.6). Measurements of osteoactivin in bone marrow and peripheral blood correlated significantly (ρ 0.59; p<0.001). Additionally, we found significant differences of osteoactivin levels between patients and control person in peripheral blood (p = 0.03).

### Prognostic value of soluble TIE2 and osteoactivin in bone marrow of MGUS vs. NDMM patients

**Fig 2:**
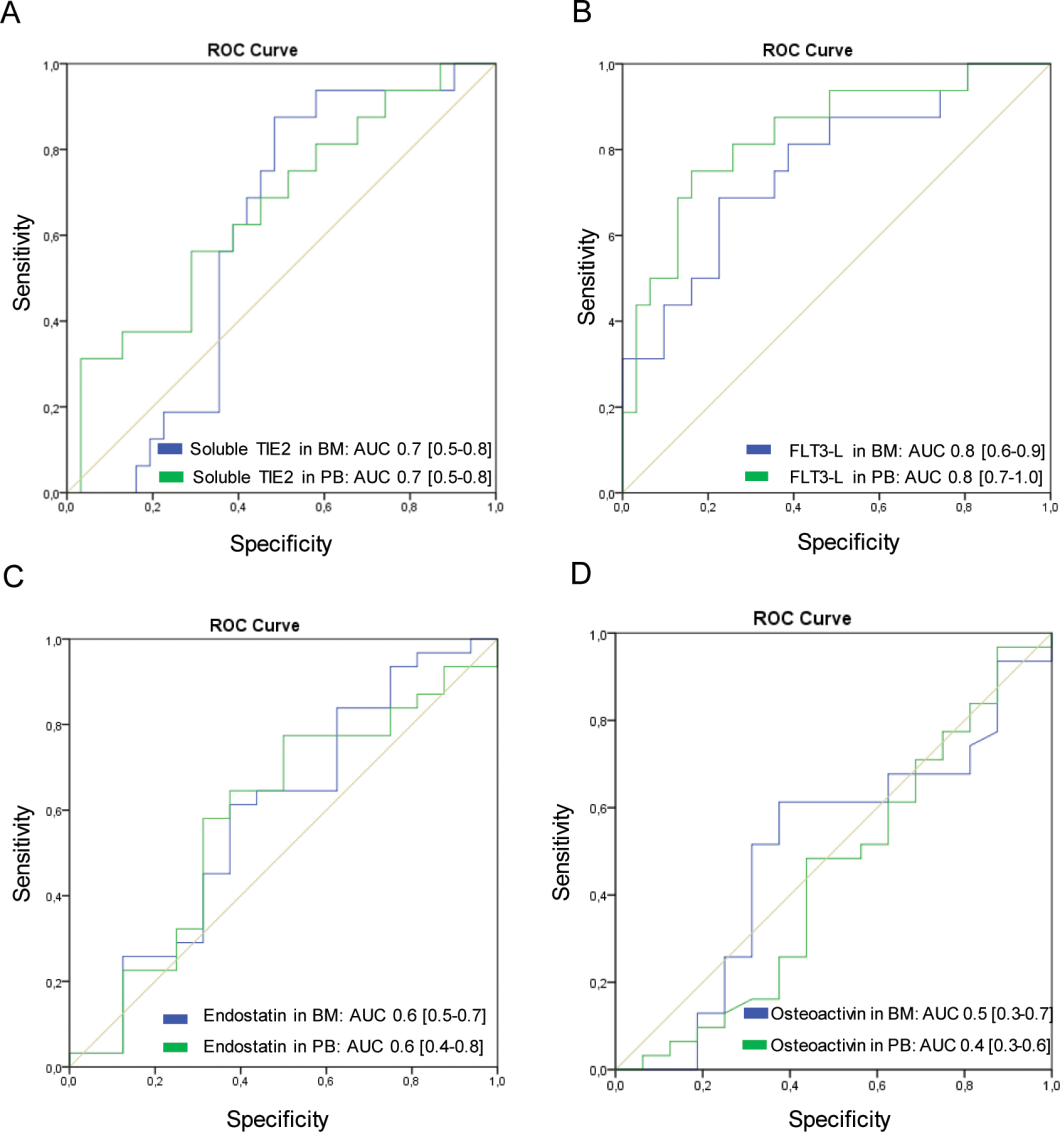
Receiver Operating Curve (ROC) analyses for bone marrow- (BM) and peripheral blood (PB) plasma soluble TIE2, FLT3-L, endostatin and osteoactivin to differentiate MGUS, NDMM and RRMM patients. (A) Soluble TIE2. (B) FLT3-L. (C) Endostatin. (D) Osteoactivin. AUC, Area under the Curve; BM, bone marrow; PB, peripheral blood.

To evaluate bone marrow plasma levels of soluble TIE2 and osteoactivin as a MM prognostic marker, we calculated the Receiver Operating Curve (ROC) by plotting sensitivity against specificity for soluble TIE2 and osteoactivin in MGUS and NDMM patients. We found that bone marrow plasma levels of soluble TIE2 and osteoactivin can discriminate MGUS- from NDMM patients (soluble TIE2: AUC = 0.8, 95% CI: 0.67–0.94; osteoactivin: AUC = 0.76, 95% CI: 0.61–0.91). An optimal cut-off value for soluble TIE2 (<3753 pg/ml) and osteoactivin (<28.7 ng/ml), which is of critical importance to accurate NDMM diagnosis, was determined by the score closest to the value under peak sensitivity (soluble TIE2: 91%; osteoactivin: 76%) and specificity (soluble TIE2: 71%; osteoactivin: 79%) (Fig 2A and 2D), also called Youden Index [29]. Based on a point prevalence for RRMM of 39% (27 patients out of 69), the corresponding predictive values for soluble TIE2 yielded 91% (positive predictive value; ppV) and 71% (negative predictive value; npV), respectively. Regarding osteoactivin we found 91% for ppV and 52% for npV (Table 4A).

**Table 4:** Receiver Operating Curve analyses (ROC) and Area under the Curve (AUC) for bone marrow- (BM) and peripheral blood (PB) plasma soluble TIE2, FLT3-L, endostatin and osteoactivin in MGUS, NDMM and RRMM patients.

	Soluble TIE2	FLT-3	Endostatin	Osteoactivin
**A** **MGUS vs. NDMM (BM)** (AUC [95% CI])	**0.8 [0.67–0.94];** **P = 0.001**	0.45 [0.27–0.63]; P<0.6	0.56 [0.38–0.74]; P = 0.5	**0.76 [0.61–0.91]; P = 0.004**
optimal Threshold / Cut-off value	pos if < 3753 pg/ml	n.c	n.c	pos if < 28.7 ng/ml
Sens / Spec	91% / 71%	n.c	n.c	76% / 79%
ppV /npV	91% / 71%	n.c	n.c	91% / 52%
**B** **NDMM vs. RRMM (BM)** (AUC [95% CI])	**0.66 [0.52–0.79]; P = 0.03**	**0.75 [0.63–0.87]; P<0.001**	0.6 [0.46–0.74]; P = 0.2	0.56 [0.42–0.71]; P = 0.4
optimal Threshold / Cut-off value	n.c	pos if > 92 pg/ml	n.c	n.c
Sens / Spec	n.c	70% / 71%	n.c	n.c
ppV /npV	n.c	61% / 79%	n.c	n.c
**C** **NDMM vs. RRMM (PB)** (AUC [95% CI])	0.66 [0.50–0.81]; P = 0.07	**0.84 [0.71–0.96]; P<0.001**	0.59 [0.41–0.77]; P = 0.3	0.45 [0.26–0.63]; P = 0.5
optimal Threshold / Cut-off value	n.c	pos if > 121 pg/ml	n.c	n.c
Sens / Spec	n.c	76% / 84%	n.c	n.c
ppV /npV	n.c	71% / 87%	n.c	n.c

ROC, Receiver Operating Curve; AUC, Area under the Curve; Spec, Specifity; Sens, Sensitivity; positive predictive value, ppV; negative predictive value, npV; MGUS, monoclonal gammopathy undetermined significance; NDMM, newly diagnosed multiple myeloma; RRMM, relapsed refractory multiple myeloma; [95% CI], Confidence interval; n.c., not calculated. Threshold, sensitivity/specifity and predictive value were calculated for signifycant Area under the Curves (AUCs) using Youden's approach.

### Predictive value of FLT3-L in bone marrow and peripheral blood of NDMM- vs. RRMM patients

Bone marrow plasma levels of FLT3-L were examined by ROC analysis (FLT3-L: AUC = 0.75, 95% CI: 0.63–0.87). An optimal cut-off value of FLT3-L (>92 pg/ml) was determined by the score closest to the value under peak sensitivity (FLT3-L: 70%) and specificity (FLT3-L: 71%), as a threshold to partition the MM patients into two groups: high bone marrow plasma levels of FLT3-L (FLT3-L >92 pg/ml) and low bone marrow plasma levels of FLT3-L (FLT3-L ≤92 pg/ml) (Fig 2B). We found high FLT3-L levels to be associated with relapse or acquired resistance in MM patients. The probability of positive tested patients for relapsed or refractoriness MM was 61% (ppV), whereas a FLT3-L level ≤92 pg/ml indicated a non-relapsed or non-refractory setting with 79% probability (npV) in the study population (Table 4B). In addition, the ROC analysis of FLT3-L levels in peripheral blood revealed a high predictive power (AUC = 0.84, 95% CI: 0.71–0.96; Fig 2B). Based on a cut-off value >121 pg/ml we found a sensitivity of 76% and a specificity of 84%. Using plasma levels in peripheral blood positive tested patients face a chance of 71% (ppV) for relapse and refractoriness. The likelihood for patients with values <121 pg/ml was 87% for a non-relapsed or non-refractory setting (Table 4C).

## Discussion

In myeloma patients, diverse angiogenic factors such as VEGF, endoglin, TNF-alpha, HGF, FGF, endostatin, thrombospondin-1 (TSP-1), and angiostatin factor are already well studied in peripheral blood and bone marrow [18, 30].

Hitherto, there are no reports about soluble TIE2, FLT3-ligand (FLT3-L), endostatin, and osteoactivin levels in bone marrow blood samples of patients with different stages of myeloma disease (MGUS, NDMM, RRMM). Therefore, we analyzed prognostic significance of these factors.

High levels of FLT3-L in bone marrow and peripheral blood are associated with disease progression and a threshold of >92 pg/ml in bone marrow and > 121 pg/ml in peripheral blood identifies relapsed or refractory MM patients. Our findings are in line with the results of Kokonozaki and co-workers, who described positive correlation of soluble FLT3-L in peripheral blood with the angiogenic process in MM and additional effects on disease activity. Moreover, they observed higher levels of soluble FLT3-L in bone marrow of active MM patients (pre-treatment) in comparison to post-treatment patients [18]. In addition, in our study we observed highest FLT3-L levels in RRMM patients.

FLT3-L is involved in hematopoiesis and plays an important role in expansion and differentiation of progenitor cells in the stem cell compartment. Responding to bone marrow stress, increased FLT3-L may participate in progenitor cell expansion [17]. FLT3-L is produced by endothelial cells first as a membrane-bound and, after proteolysis, as soluble molecule [15, 31]. Bone marrow microvessels and stromal cells play an important role as source for FLT3-L [32]. High levels of FLT3-L in RRMM patients could be caused by a higher endothelial-, perivascular-, and plasma cell infiltration in these patients, resulting in higher secretion of angiogenic molecules, including cyto- and chemokines, and due to an increased interaction of plasma cells with bone marrow microenvironment [33–35]. Angiogenic process or progression of MM might be affected or provoked by FLT3-L.

In our study, expression of soluble TIE2 in bone marrow differs significantly between MGUS-, NDMM- and RRMM patients. The angiopoietin receptor TIE2 has antiangiogenic properties supporting endothelial cell survival and stabilization of blood vessels [36]. Soluble TIE2 promotes angiogenesis and vascular remodeling inhibiting angiopoietin-mediated TIE2 activation [14]. As previously described for FLT3-L, high levels of soluble TIE2 in RRMM patients might originate from the same processes. However, soluble TIE2 levels were elevated even in MGUS patients. We found that bone marrow plasma levels of TIE2 can discriminate MGUS- from NDMM patients. MGUS, with much lower density of blood vessels in bone marrow is a “preangiogenic” condition, whereas angiogenesis is a hallmark of MM progression [37, 38]. Babarovic et al. determined a significant positive correlation between VEGF and angiogenic parameters in the MGUS stage of the disease, suggesting a possible key role of VEGF in the induction of angiogenesis in early-stage disease [39]. Soluble TIE2 originates from VEGF induced cleavage and shedding of TIE2 via PI3K/Akt-dependent pathway. This could be a possible mechanism of inhibition of vascular stabilization by VEGF and subsequent promotion of vascular remodeling and angiogenesis [14], explaining high soluble TIE2 levels in MGUS patients In contrast to promotion of angiogenesis, high soluble TIE2 and subsequent block of TIE2 receptor activation can also result in blood vessel regression [40]. Therefore, strong increase of soluble TIE2 in MGUS patients might alternatively be responsible for block of angiogenesis. Opposite, modest increase of soluble TIE2 observed in RRMM patients can provoke blood vessel instability and neoangiogenesis contributing to the disease progression. Thus, further studies on origin and effects of soluble TIE2 in MGUS and MM patients are needed.

Endostatin is an endogen inhibitor of angiogenesis which interacts with endothelial cell molecules, induces apoptosis by inhibiting anti-apoptotic proteins Bcl-2 and Bcl-xL and inhibits tumor growth [22, 41]. We detected the lowest expression of endostatin in bone marrow plasma samples of RRMM patients, similar to Urbanska et al. who observed higher levels of endostatin in serum of venous blood samples of MM patients at diagnosis in comparison to MM patients after treatment [42].

The fourth evaluated marker was osteoactivin with highest expression in the MGUS cohort. Osteoactivin induces osteoblastogenesis and inhibits osteoclastogenesis mediated through CD44-ERK signaling [25]. We assume that the lower protein levels of osteoactivin in NDMM and RRMM contribute to more bone destruction and formation of osteolytic bone lesions. Plasma cells are able to express markers of osteoclasts and to activate the β3 transcriptional pathway leading to ERK1/2 phosphorylation. Thus, an uncontrolled activity of osteoclast differentiation favors the development and the progress of osteolytic lesions in myeloma bone disease [43]. As previously described, myeloma progression is characterized by neoangiogenesis [44]. Although reported as proangiogenic factor in breast cancer, low levels of osteoactivin in RRMM suggest its insignificant role in promotion of angiogenesis in MM. Only, high levels of osteoactivin in MGUS correlate with above mentioned induction of angiogenesis in this stage of the disease. Therefore, our results indicate stronger association between osteoactivin and osteogenesis than between osteoactivin and angiogenesis in MGUS and MM.

In summary, our study is the first report analyzing the prognostic significance of four different biomarkers in plasma samples of bone marrow and peripheral blood in three different MM cohorts. We found high FLT3-L levels in bone marrow and peripheral blood (cut-off value/threshold of FLT3-L >92 pg/ml in bone marrow and >121 pg/ml in peripheral blood) to be associated with relapse or refractoriness in MM patients. Based on significantly higher expression of FLT3-L in RRMM patients than in MGUS and NDMM patients, our data suggest that FLT3-L could be useful as marker to identify RRMM patients and should be evaluated as potential target for future therapy strategies.
